# The Alkaline Trap: Severe Calcium-Alkali Syndrome Secondary to Chronic Calcium Carbonate Ingestion

**DOI:** 10.7759/cureus.116335

**Published:** 2026-09-16

**Authors:** Jasmine Kaur, Moieze R Malik, Neharika Narayanan, Jovitta Fernandez Jerrin, Cornelius Fernandez

**Affiliations:** 1 Oncology, Pilgrim Hospital, Boston, GBR; 2 Medicine, Pilgrim Hospital, Boston, GBR; 3 Medicine, University of Leicester, Boston, GBR; 4 Endocrinology, United Lincolnshire Hospitals NHS Trust, Lincoln, GBR

**Keywords:** calcium-alkali syndrome, calcium carbonate, hypercalcaemia, metabolic alkalosis, milk-alkali syndrome, peripheral neuropathy, pth-independent hypercalcaemia, rennie, vitamin b12 deficiency, vitamin d deficiency

## Abstract

Calcium-alkali syndrome is an increasingly recognised cause of parathyroid hormone (PTH)-independent hypercalcaemia resulting from excessive calcium and absorbable alkali ingestion, commonly through calcium-containing antacids or supplements. Its clinical presentation may mimic neurological or malignant disease, potentially leading to extensive investigation and delayed diagnosis. A 64-year-old woman presented following a fall with paraparesis and lower back pain. Investigations demonstrated severe hypercalcaemia (adjusted calcium 4.83 mmol/L), suppressed PTH of 1.4 pmol/L, metabolic alkalosis (pH 7.47; bicarbonate 39.8 mmol/L), and mild renal impairment. Extensive investigation for malignant and granulomatous causes was unremarkable. Neurological assessment identified bilateral symmetrical peripheral neuropathy, and severe vitamin B12 deficiency was subsequently identified. Following intravenous fluid resuscitation and a single dose of pamidronate, serum calcium fell to 1.69 mmol/L. Subsequent testing demonstrated severe vitamin D deficiency with an appropriately elevated PTH. Detailed medication review revealed ingestion of 6-12 Rennie® tablets daily for more than two years for chronic indigestion. In the context of excessive calcium carbonate exposure and the characteristic biochemical triad of hypercalcaemia, metabolic alkalosis, and renal impairment, calcium-alkali syndrome was diagnosed. This case highlights the importance of obtaining a detailed over-the-counter medication history when evaluating severe hypercalcaemia. Early recognition of calcium-alkali syndrome can prevent unnecessary investigations, diagnostic delay, and avoidable morbidity.

## Introduction

Hypercalcaemia can be categorised as parathyroid hormone (PTH)-dependent or PTH-independent based on the PTH levels. The conditions associated with PTH-dependent hypercalcaemia include primary hyperparathyroidism (PHPT), lithium-associated hyperparathyroidism, familial hypocalciuric hypercalcaemia (also known as familial benign hypercalcaemia) and tertiary hyperparathyroidism [1].

The conditions associated with PTH-independent hypercalcaemia include malignancy and vitamin D-related hypercalcaemia (vitamin D intoxication or granulomatous diseases), calcium-alkali syndrome (previously known as milk-alkali syndrome), endocrine causes (thyrotoxicosis, adrenal insufficiency and rarely phaeochromocytoma), drugs (thiazide diuretics and vitamin A), and miscellaneous causes like immobilisation [1].

PHPT and malignancy together account for 80%-90% of all cases. In ambulant patients with hypercalcaemia, PHPT accounts for 60% of cases, whereas in hospitalised patients with hypercalcaemia, malignancy accounts for 54%-65% of cases [2]. Calcium-alkali syndrome has been reported to account for approximately 9%-12% of hypercalcaemia cases in some hospital-based cohorts, making it the third most common cause of hypercalcaemia after PHPT and malignancy; however, the reported prevalence varies according to the population studied and contemporary calcium supplementation practices [3].

Clinical manifestations of calcium-alkali syndrome can vary and may include gastrointestinal symptoms, acute kidney injury, neurocognitive impairment, muscle weakness and cardiac abnormalities [1,3]. Consequently, calcium-alkali syndrome may mimic hypercalcaemia associated with malignancy, granulomatous disease and PHPT, which mostly results in extensive investigation before the diagnosis is established [4-6].

We report a case of severe calcium-alkali syndrome secondary to chronic calcium carbonate ingestion presenting with severe hypercalcaemia, recurrent falls, gait instability, muscle weakness, together with metabolic alkalosis and mild renal impairment. The coexistence of severe hypercalcaemia and vitamin B12 deficiency contributed to the diagnostic challenge and highlighted the importance of considering multiple concurrent pathologies when assessing patients presenting with hypercalcaemia and neurological symptoms.

## Case presentation

A 64-year-old woman presented following a fall with progressive lower-limb weakness, paraparesis and lower back pain. She reported recurrent falls and worsening mobility over several months. There was no history of weight loss, constitutional symptoms or other features suggestive of malignancy.

Initial investigations demonstrated severe hypercalcaemia with an adjusted serum calcium concentration of 4.83 mmol/L (reference range: 2.20-2.60 mmol/L), normal phosphate of 1.0 mmol/L and a suppressed PTH concentration of 1.4 pmol/L, consistent with PTH-independent hypercalcaemia. Additionally, hypomagnesaemia (0.49 mmol/L) was noted with associated hypokalaemia (2.5 mmol/L) (Table 1).

**Table 1:** Biochemical findings on admission

Parameter	Admission	Reference range
Adjusted calcium (mmol/L)	4.83	2.2-2.6
Phosphate (mmol/L)	1.00	0.8-1.5
Parathyroid hormone (PTH) (pmol/L)	1.4	1.6-6.9
Magnesium (mmol/L)	0.49	0.7-1.0
Potassium (mmol/L)	2.5	3.5-5.3
Urea (mmol/L)	8.9	2.5-7.8
Creatinine (μmol/L)	94	45-84
Glomerular filtration rate (GFR) (EPI-2009) (mL/min)	70	90-200

Venous blood gas (VBG) analysis revealed metabolic alkalosis with a pH of 7.466 and bicarbonate of 39.8 mmol/L (Table 2). Mild renal impairment relative to baseline was noted, with serum creatinine of 94 μmol/L, urea of 8.9 mmol/L and a reduction in estimated glomerular filtration rate (70 mL/min) (baseline renal function: eGFR > 90 mL/min) (Table 1).

**Table 2:** Venous blood gas (VBG) findings on admission

Parameters	Findings	Reference range
pH	7.466	7.320-7.430
Actual bicarbonate (mmol/L)	39.8	22.0-29.0

Due to hypercalcaemia with suppressed PTH, investigations for malignancy and granulomatous diseases were carried out. Computed tomography of the neck, chest, abdomen and pelvis demonstrated no evidence of malignancy. Myeloma screening was negative. Serum protein electrophoresis demonstrated no monoclonal band, and serum free light chain analysis (including kappa and lambda free light chains and kappa/lambda ratio) showed no evidence of a plasma cell dyscrasia. Breast and skin examination showed no evidence of malignancy. Serum angiotensin-converting enzyme levels were low (<12).

Neurological assessment identified bilateral symmetrical peripheral neuropathy. Magnetic resonance imaging of the spine excluded compressive myelopathy or other structural neurological pathology (Figure 1). Further investigations revealed severe vitamin B12 deficiency with a serum concentration of 111 ng/L (180-771), with normal folate and ferritin and negative gastric parietal and intrinsic factor antibodies. Vitamin B12 replacement was initiated.

**Figure 1:**
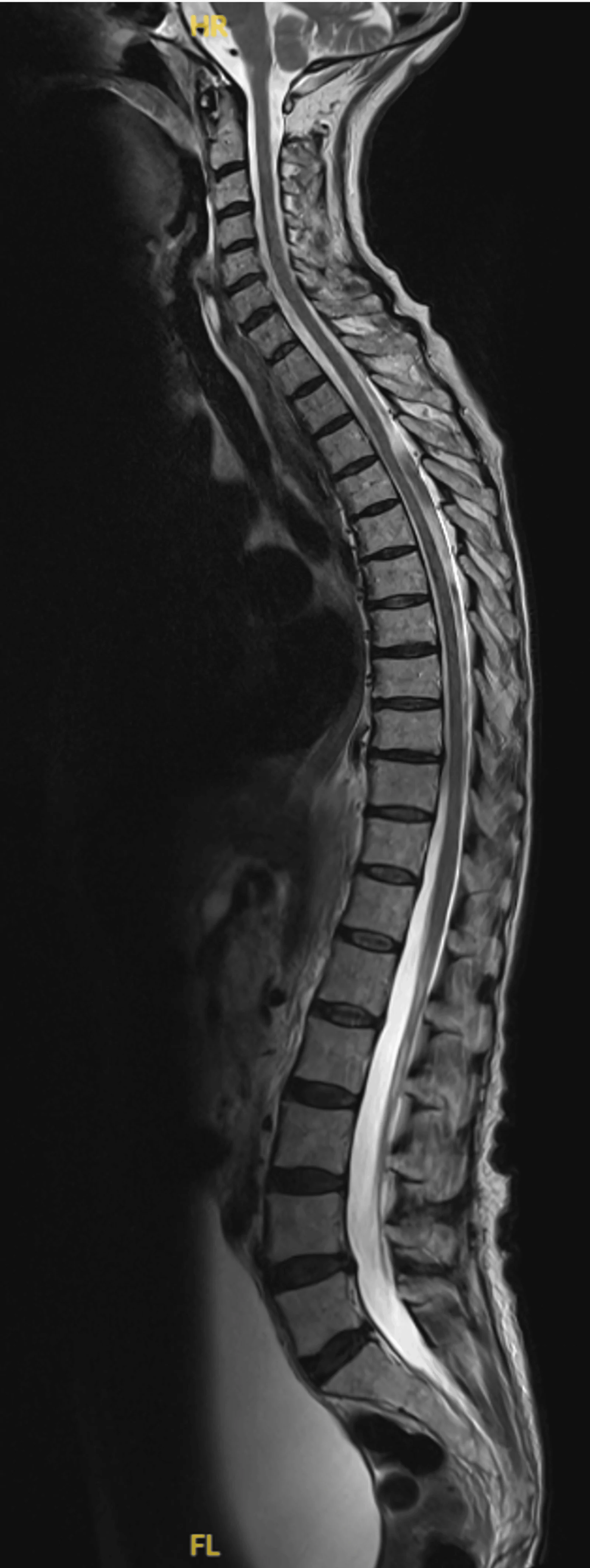
Sagittal T2-weighted MRI of the whole spine showing no evidence of compressive myelopathy Sagittal T2-weighted magnetic resonance imaging of the whole spine was performed during the investigation of progressive lower-limb weakness and recurrent falls. No evidence of spinal cord compression, compressive myelopathy, spinal canal stenosis or other significant structural spinal pathology was identified.

The patient received intravenous 0.9% sodium chloride for volume repletion followed by a single 90 mg dose of intravenous pamidronate on admission. Serum calcium normalised within three days of treatment; however, the corrected calcium subsequently fell to a nadir of 1.69 mmol/L 10 days after pamidronate administration. Intravenous 10% calcium gluconate replacement was given, resulting in normalisation of serum calcium within 24 hours. Biochemical testing at that time revealed severe vitamin D deficiency with 25-hydroxyvitamin D levels of 10 nmol/L and an appropriately elevated PTH concentration of 18.8 pmol/L. Coeliac serology and oesophagogastroduodenoscopy did not identify an alternative cause for malabsorption.

A detailed medication review ultimately revealed consumption of 6-12 Rennie® tablets daily for over two years for chronic indigestion. Each Rennie® tablet contains 680 mg calcium carbonate (equivalent to 272 mg elemental calcium). This corresponded to an estimated daily calcium carbonate intake of 4.1-8.2 g and elemental calcium intake of approximately 1.6-3.3 g per day. In the context of excessive calcium carbonate exposure along with resultant severe hypercalcaemia, metabolic alkalosis and renal impairment, a diagnosis of calcium-alkali syndrome was established.

Calcium carbonate was discontinued, and vitamin D and B12 replacement therapy continued. The patient's biochemical abnormalities resolved, and her neurological symptoms gradually improved with treatment. Blood tests done 15 days after admission showed an adjusted calcium of 2.46 mmol/L, phosphate of 0.82 mmol/L, and PTH of 4.6 pmol/L, indicating that the suppressed PTH during the previous hypercalcaemia was an appropriate physiological response rather than an autonomous pathological process.

## Discussion

Calcium-alkali syndrome (previously known as milk-alkali syndrome) remains an important and under-recognised cause of severe hypercalcaemia. Although historically linked to milk and alkali regimens used for peptic ulcer disease, modern cases are predominantly associated with excessive use of over-the-counter or prescribed calcium carbonate-containing antacids or osteoporosis supplements containing calcium carbonate and vitamin D [3,5,7]. The increased availability and consumption of these calcium carbonate preparations have contributed to the resurgence of the condition, which now represents a significant proportion of cases of non-PTH-mediated hypercalcaemia [5,7].

When calcium carbonate dissolves in the stomach, the carbonate content is absorbed as bicarbonate equivalents, producing a net alkali load. Characteristic biochemical abnormalities of calcium-alkali syndrome arise from excessive ingestion of calcium and absorbable alkali. The pathophysiology of calcium-alkali syndrome involves excessive calcium intake. Hypercalcaemia results in renal vasoconstriction, reduced glomerular filtration, natriuresis and volume depletion. Volume depletion acts with alkalosis on the proximal tubules to enhance bicarbonate reabsorption, worsening the metabolic alkalosis, and passive paracellular calcium reabsorption, worsening the hypercalcaemia. The alkali ingestion promotes metabolic alkalosis and increases distal tubular active calcium reabsorption, further augmenting hypercalcaemia, creating a perpetuating cycle of hypercalcaemia and renal dysfunction [3,8]. The complex interplay between hypercalcaemia, metabolic alkalosis and renal dysfunction is illustrated in Figure 2, and the schematic representation of molecular-level changes happening at various parts in the nephron is given in Figure 3.

**Figure 2:**
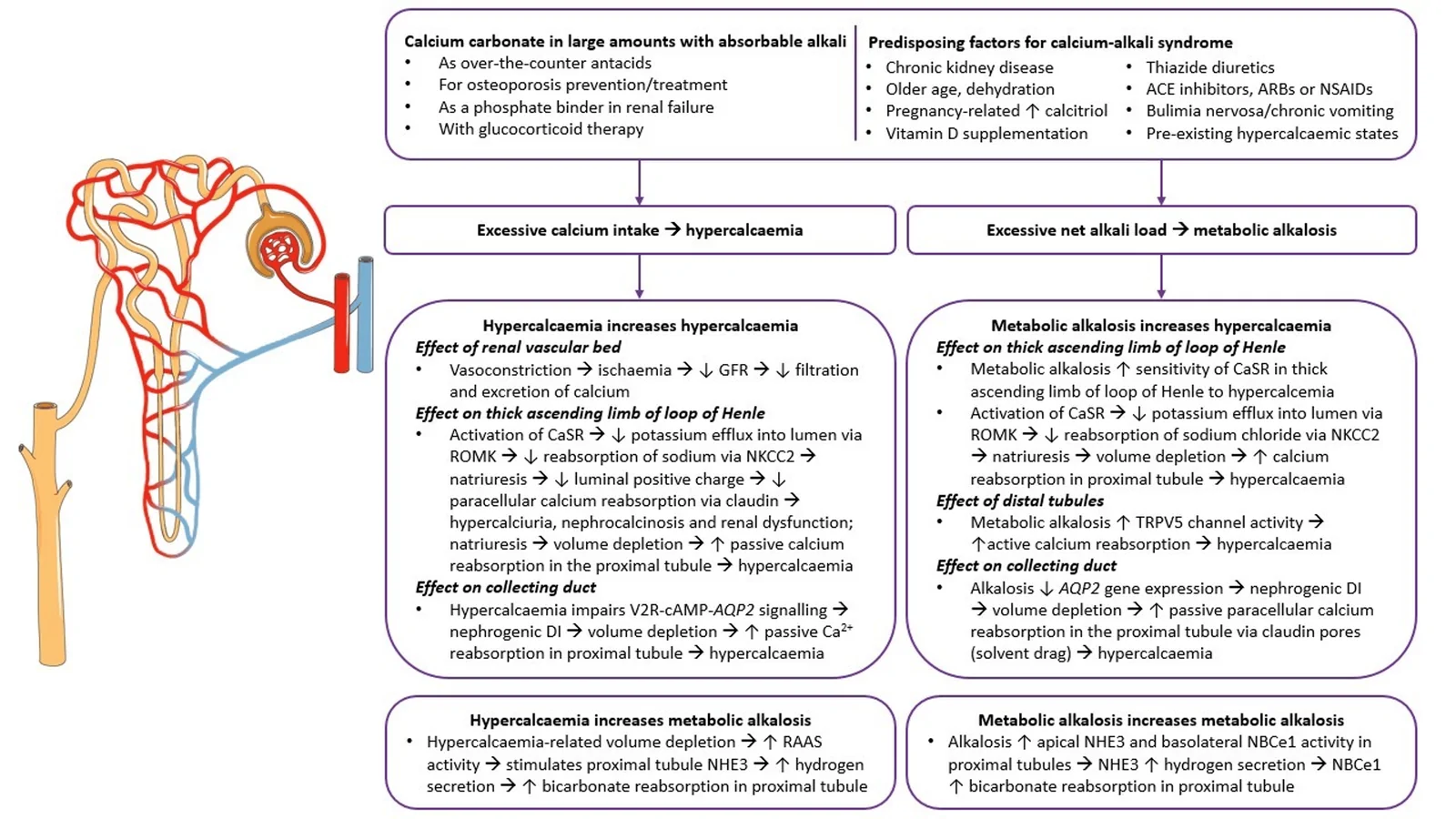
Pathophysiology of calcium-alkali syndrome illustrating the self-perpetuating cycle between excessive calcium carbonate ingestion, hypercalcaemia, metabolic alkalosis and renal dysfunction ACE: Angiotensin-converting enzyme; AQP2: Aquaporin-2; ARB: Angiotensin receptor blocker; cAMP: Cyclic adenosine monophosphate; CaSR: Calcium-sensing receptor; DI: Diabetes insipidus; GFR: Glomerular filtration rate; NBCe1: Electrogenic sodium-bicarbonate cotransporter 1; NHE3: Sodium-hydrogen exchanger 3; NKCC2: Sodium-potassium-2 chloride cotransporter 2; NSAID: Non-steroidal anti-inflammatory drug; RAAS: Renin-angiotensin-aldosterone system; ROMK: Renal outer medullary potassium channel; TRPV5: Transient receptor potential vanilloid 5 calcium channel; V2R: Vasopressin V2 receptor. Image credit: This is an original figure created by Dr Cornelius Fernandez James using Microsoft PowerPoint (Microsoft Corporation, Redmond).

**Figure 3:**
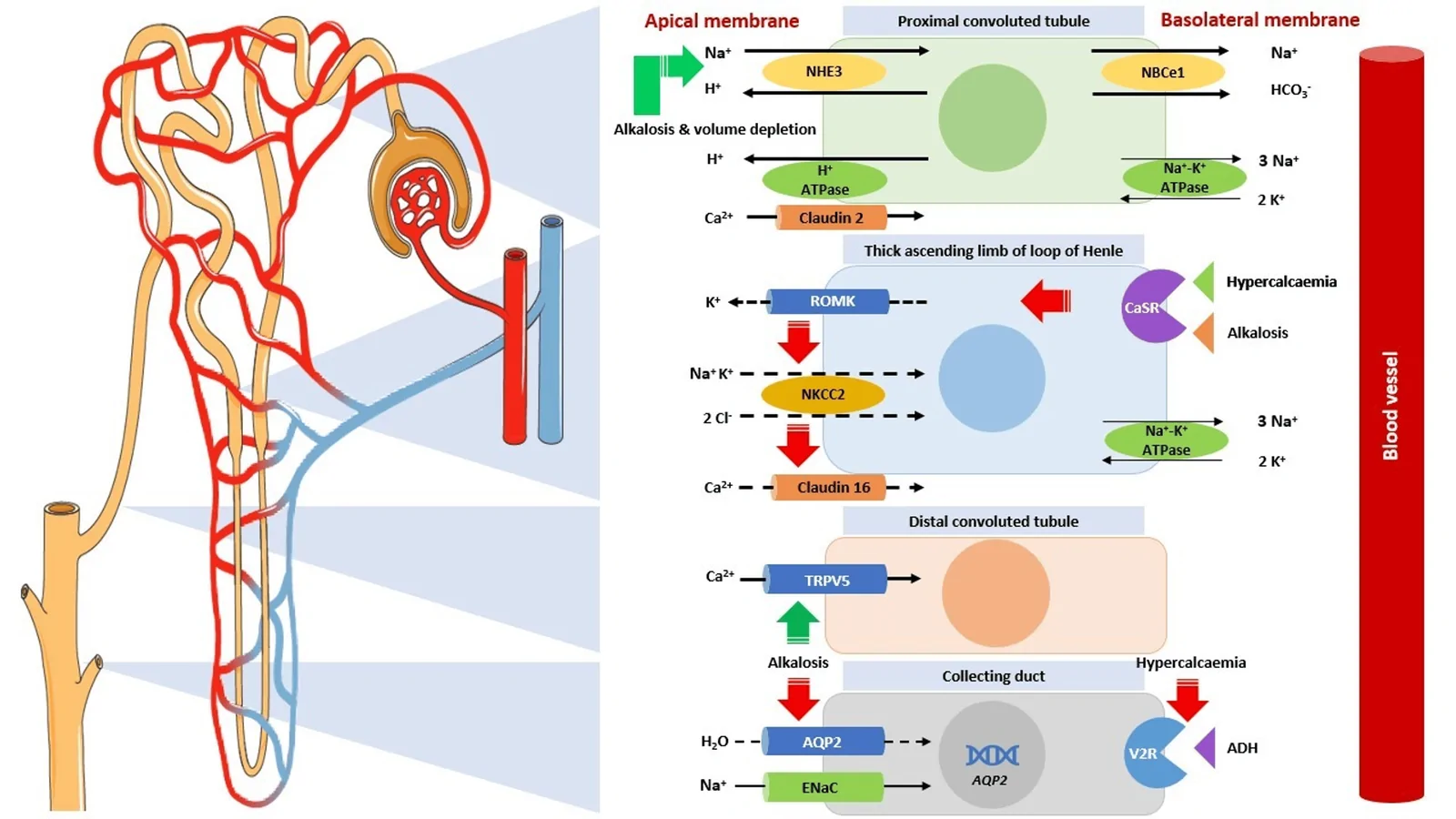
Pathophysiology of calcium-alkali syndrome illustrating the molecular-level changes occurring at various sites within the nephron during hypercalcaemia and metabolic alkalosis Physiological concepts were informed by Patel et al. [9]. Dashed arrows indicate decreased transport, whereas solid arrows indicate increased transport across ion channels. Green arrows indicate activation, whereas red arrows indicate inhibition of downstream molecular processes. ADH: Antidiuretic hormone; AQP2: Aquaporin-2; ATPase: Adenosine triphosphatase; CaSR: Calcium-sensing receptor; ENaC: Epithelial sodium channel; NBCe1: Sodium-bicarbonate cotransporter 1; NHE3: Sodium-hydrogen exchanger 3; NKCC2: Sodium-potassium-2 chloride cotransporter 2; ROMK: Renal outer medullary potassium channel; TRPV5: Transient receptor potential vanilloid 5 calcium channel; V2R: Vasopressin V2 receptor. Image credit: This is an original figure created by Dr Cornelius Fernandez James using Microsoft PowerPoint (Microsoft Corporation, Redmond, WA).

Our patient demonstrated the classical triad of calcium-alkali syndrome, presenting with severe hypercalcaemia, metabolic alkalosis and renal impairment, aligning with the diagnosis. An adjusted calcium concentration of 4.83 mmol/L represents life-threatening hypercalcaemia and prompted investigation to exclude malignancy, myeloma and granulomatous disease [4]. Despite the presence of the classical triad, the diagnosis was not established until further detailed medication history was undertaken and revealed chronic excessive calcium carbonate intake. Previous studies have highlighted that calcium-alkali syndrome is frequently overlooked because patients often fail to report over-the-counter medications unless specifically questioned [5,6]. This case emphasises the importance of obtaining a comprehensive medication history in all patients undergoing investigation for hypercalcaemia.

Severe hypercalcaemia is associated with neuromuscular weakness, impaired gait, altered cognition and reduced functional status [1]. The patient's history of frequent falls, muscle weakness, and impaired gait was likely attributable to profound hypercalcaemia. Improvement following correction of hypercalcaemia supports a direct contribution from the metabolic disturbance.

The hypomagnesaemia in this case was likely multifactorial, with contributions from metabolic alkalosis, hypercalcaemia and suppressed PTH, all causing reduced renal tubular reabsorption and thereby increased renal excretion of magnesium. The pathogenesis of hypokalaemia in this case was secondary to hypomagnesaemia and metabolic alkalosis, as well as increased renal excretion of potassium from severe hypercalcaemia.

The diagnostic challenge in this case arose from the coexistence of severe hypercalcaemia and vitamin B12 deficiency-associated peripheral neuropathy, both of which likely contributed to the patient's neurological symptoms. Gastric acidity plays an essential role in the release of food-bound cobalamin before intestinal absorption, and states of reduced gastric acidity have been associated with vitamin B12 deficiency [10]. Chronic ingestion of large quantities of calcium carbonate may have reduced gastric acidity and could have contributed to impaired release of food-bound cobalamin, although a direct causal relationship cannot be established in this case. The associated peripheral neuropathy likely compounded the neurological effects of severe hypercalcaemia and contributed to the patient's falls and lower-limb weakness. Reports describing the coexistence of calcium-alkali syndrome and significant vitamin B12 deficiency are limited, making this association noteworthy.

The endocrine features also highlight an important diagnostic principle. Hypercalcaemia suppresses PTH secretion and therefore can mask concurrent abnormalities of calcium homeostasis [11]. PTH was appropriately suppressed during severe hypercalcaemia but became elevated following correction of hypercalcaemia. Relief of calcium-mediated suppression of PTH secretion, together with severe vitamin D deficiency, resulted in secondary hyperparathyroidism. At the time, the PTH concentration was 18.8 pmol/L, and the adjusted serum calcium concentration was 1.96 mmol/L. This elevated PTH level was therefore considered an appropriate physiological response to hypocalcaemia in the setting of severe vitamin D deficiency, which became apparent following correction of the preceding hypercalcaemia.

Management of calcium-alkali syndrome focuses on removal of the offending calcium and alkali source along with intravenous volume repletion [3,8]. In this case, pamidronate was administered because of the profound hypercalcaemia (adjusted calcium 4.83 mmol/L) and associated clinical manifestations. Although bisphosphonates are occasionally used in severe cases, caution is advised because significant hypocalcaemia may develop once the underlying disorder resolves, particularly in the presence of vitamin D deficiency [3,8]. Severe vitamin D deficiency was subsequently identified in our patient and likely contributed to the development of post-treatment hypocalcaemia (calcium nadir 1.69 mmol/L) by limiting the physiological ability to maintain serum calcium concentrations following suppression of bone resorption.

This case is notable for the constellation of profound hypercalcaemia, unmasked vitamin D deficiency upon bisphosphonate treatment, dynamic PTH changes during therapy, coexisting vitamin B12 deficiency contributing to peripheral neuropathy, coexisting vitamin B12 and D deficiency raising the suspicion of malabsorption and associated low magnesium/potassium with metabolic alkalosis. Together, these factors increased the diagnostic complexity and outlined the value of a broad and systematic approach to investigation.

## Conclusions

This case highlights calcium-alkali syndrome as an important and potentially reversible cause of severe PTH-independent hypercalcaemia. Excessive consumption of over-the-counter calcium carbonate-containing antacids resulted in profound hypercalcaemia, metabolic alkalosis, renal impairment and significant neuromuscular weakness. The coexistence of vitamin B12 deficiency and severe vitamin D deficiency contributed to the clinical presentation and complicated interpretation of biochemical findings. Recognition of the characteristic biochemical triad and thorough enquiry regarding non-prescription medication use are essential for avoiding diagnostic delay, unnecessary investigations and preventable morbidity.
